# Single standard calibration and data processing in flow injection titration based on concentration gradients

**DOI:** 10.1155/S1463924697000163

**Published:** 1997

**Authors:** M. C. U. Araújo, A. V. Santos, R. S. Honorato, C. Pasquini

**Affiliations:** 1 Departamento de Química, CCEN Universidade Federal da Paraíba C.P. 5093 João Pessoa PB 58051-970 Brazil; 2 Instituto de Quimica Universidade Estadual de Campinas Brazil

## Abstract

This paper describes use of gradients of concentration generated in flow injection (FI) systems to perform determinations based on points where the concentration of titrant and analyte are at stoichiometric ratio. Two procedures were developed. In one procedure the titrant is injected in a FI manifold and merges with the sample which is continuously pumped towards the detector. In the other procedure the sample is injected and merged with the titrant which is continuously pumped. Both techniques make use of concentration gradients of the sample or titrant generated in FI manifolds that contain a mixing chamber. This gradient is calibrated employing only one standard solution (usually the titrant) in order to convert any detector signal, obtained in the elapsed time after injection, to instantaneous concentration values. The flow system is microcomputer controlled and data are treated to locate points where the concentration of titrant and analyte are at the stoichiometric ratio. These points are found in abrupt changes of the signal × concentration curves obtained in the presence of the reaction. The method has been evaluated for determination of Fe(II) and acetic acid by spectrophotometric and conductimetric detection, respectively. Results show a mean relative standard deviation lower than 1%, an average accuracy of 1% and a high sampling processing capability (40 to 60 samples per hour).


Journal of Automatic Chemistry, Vol. 19, No. 5 (September–October 1997) pp. 157–164

### Single standard calibration and data processing in flow injection titration based on concentration gradients

**M. C. U. Araújo, A. V. Santos, R. S. Honorato**

*Departamento de Química, CCEN, Universidade Federal da Paraíba, C.P. 5093, 58051-970, João Pessoa, PB, Brazil*

**and C. Pasquini**

*Instituto de Química, Universidade Estadual de Campinas*

*This paper describes use of gradients of concentration generated in flow injection (FI) systems to perform determinations based on points where the concentration of titrant and analyte are at stoichiometric ratio. Two procedures were developed. In one procedure the titrant is injected in a FI manifold and merges with the sample which is continuously pumped towards the detector. In the other procedure the sample is injected and merged with the titrant which is continuously pumped. Both techniques make use of concentration gradients of the sample or titrant generated in FI manifolds that contain a mixing chamber. This gradient is calibrated employing only one standard solution (usually the titrant) in order to convert any detector signal, obtained in the elapsed time after injection, to instantaneous concentration values. The flow system is microcomputer controlled and data are treated to locate points where the concentration of titrant and analyte are at the stoichiometric ratio. These points are found in abrupt changes of the signal × concentration curves obtained in the presence of the reaction. The method has been evaluated for determination of Fe(II) and acetic acid by spectrophotometric and conductimetric detection, respectively. Results show a mean relative standard deviation lower than 1%, an average accuracy of 1% and a high sampling processing capability (40 to 60 samples per hour).*

### Introduction

When performed manually, titrimetric methods are cumbersome and slow. To overcome these drawbacks, automatic batch and continuous flow systems have been developed. Development of batch automatic titrators started more than 80 years ago [1] and this continues [2]. Automatic batch titration, however, requires individual flasks to contain each sample and, despite efforts to increase the speed of determination, the overall sampling processing is considered slow. Furthermore, batch procedures require mechanical units to deliver the titrant and to homogenize the titrant and sample mixture and these units are expensive.

The inconveniences of batch classical titration procedure have been minimized, employing continuous flow methods, since the classical work by Blaedel and Laessig [3]. Following this first approach, some interesting modifications have been proposed to the flow methodology [4–7]. In those systems, at least one point can be obtained when the concentration of the titrant and analyte are at the stoichiometric ratio of the reaction. Usually this point is reached by keeping the flow rate of one of the solutions (sample or titrant) constant and making the other change linearly [3–5] or by keeping the flow rate of sample constant and by exploiting linear concentration gradients of titrant generated by an external gradient chamber [6] or by coulometry [7]. Unlike conventional titration which uses the concept of equivalent quantity, the flow approach employs the concept of equivalent concentration.

With the advent of flow injection [8], a new flow procedure (FI titration) was developed [9]. A peak-like gradient of the sample is generated in a carrier flow containing a titrant. Sharp signal changes indicate two instantaneous points where the concentration of the titrant is at a stoichiometric ratio to that of the analyte in the sample. The time interval elapsed between both points is linearly related, under certain conditions, to the logarithm of the sample concentration. Calibration, employing various standardized sample solutions, is necessary to establish this relationship. To increase the sensitivity of such systems, a flow reversal FI has also been proposed [10].

Another FI technique (single-point titration [11]) has demonstrated that a linear relationship exists between the peak height and the concentration of an acid or base when a potentiometric sensor, like a glass electrode, is employed. For spectrophotometric sensors, the same situation was observed when neutralization reactions were employed and followed with the aid of acid–base indicators [12].

Although intrinsically based on the exploitation concentration gradients generated by changing the flow rate of the sample or titrant [3–5], by external gradient chamber [6] by coulometry [7] or by FI systems [9–12], all of these flow titrations rely on calibration, performed with the use up to six standardized sample solutions, to relate the analytical parameter to the sample concentration. Therefore, the direct use of stoichiometry is not found in flow techniques.

The binary searching approach has recently been applied to flow titrations [13]. This method makes use of variable volumetric ratios between the titrant and sample solution. Ratios are selected by the binary algorithm. About 10 searching steps are sufficient to estimate, with a reasonable precision, the stoichiometric ratio between titrant and analyte. The concentration of the titrant solution is employed directly and no calibration is necessary. However, this method applies only to systems where an indicator can be used by the binary searching algorithm to decide the next step in the searching process.

0142-0453/97 $12·00 © 1997 Taylor & Francis Ltd

Therefore, self-indicating titrations, as conductimetric titrations, have not been performed.

In this paper, a new flow titration methodology is proposed. The methodology uses the concentration gradient pattern generated by FI systems and is based on the FI standard addition methodology previously described [14–16]. In the flow manifold, the concentration of one of the solutions (sample or titrant) is kept at its steady state during the whole process, by pumping it continuously. The other solution is injected and its concentration is determined by the gradient calibration technique [14–16].

The methodology described makes use of Stoichiometric Ratio Points (SRP), which are instantaneous concentration points located between regions where excess of titrant and analyte occurs. At these points, the instantaneous concentration of the injected solution is at the stoichiometric ratio in relation to that of the continuous pumped solution. SRPs are found experimentally when abrupt changes are observed: these experimental points are called SREPs (Stoichiometric Ratio Experimental Points). The SREPs are found in curves when titration signals (absorbance or conductance, for example) are plotted against the concentration of the injected specimen, replacing the time points by the values of the heights of the calibration profile at that time. These values are proportional to the concentration of the injected specimen at that time and in absence of reaction. This procedure ensures that the changing property is evaluated against the instantaneous concentration of the injected specimen in a similar fashion to conventional titrations which are evaluated for end point determination in a curve of the property versus added amount of titrant.

The FI titration methodology was evaluated using spectrophotometric detection for determination of Fe(II), employing $KMnO_4$ as titrant, and for determination of total acidity in vinegar, employing a conductimetric detection and NaOH as titrant.

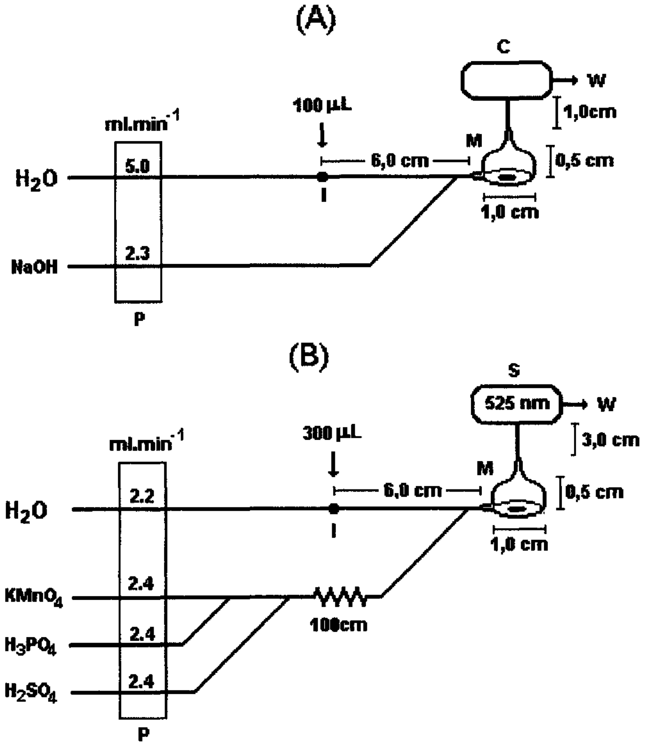

*Figure 1. FI manifolds employed by the SIT methodology. (A) manifold for conductimetric determination of acetic acid in vinegar and (B), manifold for spectrophotometric determination of Fe(II). I, injection point; P, peristaltic pump; M, mixing chamber; W, waste line; S, spectrophotometric flow cell and C, conductimetric flow cell. For TIT, the titrant solutions are replaced, in both manifolds (A) and (B), by sample solutions and the volume injected refers to that of the titrant solution.*

### Experimental

*Reagents, samples and standard solutions*

0·1 M stock solutions of $KMnO_4$, NaOH, and Fe(II) were prepared and standardized employing conventional methodology [17]. Acid solutions employed in the iron(II) titrations were $H_3PO_4$, 0·03 M and $H_2SO_4$, 0·01 M. Other solutions were prepared by suitable dilution of the stock solutions.

Samples of iron ore and alloys were treated and dissolved using well-known procedures [17]. Vinegar samples were analysed directly from the flask without any previous treatment. The reagents employed were of analytical grade and fresh distilled water free of $CO_2$ was always utilized.

*Equipment*

Flow determinations were monitored by a Radelkis-OK-102/1 conductivity meter or by a Micronal-B342-II visible spectrophotometer. The conductimetric flow cell is very simple and was made of an acrylic block (1 × 1 × 2 cm) with a 1-mm-diameter hole. Two stainless-steel screws were attached perpendicularly to the hole and at 180°. The exposed end surfaces of the screws constitute the two electrodes of the flow cell. The home-made spectrophotometric flow cell [18] was a 1-cm-long, 2 mm i.d. glass tube placed in the optical path of the spectrophotometer. Light reaches the tube perpendicularly. In the flow cells, the fluids flow from the inferior to the superior part of the tube helping to avoid air bubbles trapping inside the cell.

A peristaltic pump (Milan-206, SP-Brazil) was used; Tygon pumping tubes were employed with polyethylene tubing of 0·75 mm i.d. to assemble the FI manifolds. An automatic sample injection device [19] was used to introduce the samples in the carrier fluids. The mixing chamber (figure 1) is a mini-bottle with a 1·0 cm base diameter, 0·5 cm high and about 1 ml inner volume. Stirring was magnetic.

A microcomputer, compatible with the IBM-AT 486, was employed to control the injection device and for data acquisition using a versatile interface [20]. The interface converts the analogue signal from the detectors to a 12 bit resolution digital signal and actuates the solenoids microvalves of the injection device [19].

The control, data acquisition and data treatment program was developed in QuickBasic 4.5. The software is menu driven and provides the procedures to calibrate the concentration gradient, to locate the Stoichiometric Ratio Experimental Point, SREPs and to calculate the analyte concentration.

### *Flow manifolds*

Figure 1 depicts the two FI manifolds employed. The connections from the point where the water carrier stream merges with the titrant to the mixing chamber and from there to the flow cell, were made as short as possible. In this way, the dispersion from the merging point to the detector is determined by the mixing chamber.

Two FI titration procedures were developed: Sample Injection Titration-SIT, and Titrant Injection Titration-TIT. As shown in figure 1, the manifolds were used to perform a SIT in which a discrete volume of sample is injected and merged with the titrant that is continuously pumped towards the detector. The injected sample is dispersed, mainly by convection, in the mixing chamber, over a constant steady-state concentration of the titrant. In the TIT, a discrete volume of titrant is injected and merged with the sample which is, by this time, continuously pumped towards the detector through the tube previously occupied by the titrant ($KMnO_4$ or NaOH in the manifolds of figure 1).

### *Gradient calibration*

The gradient calibration technique previously described [14–16] is used to find the instantaneous concentration of a solution injected in the flow manifold, in absence of reaction, by associating the time elapsed from the time of injection to a dilution-like factor. This factor defines the relationship of the concentration of the original solution with the instantaneous concentration at the detection point. It means, for example, that after gradient calibration, the concentration of the injected solution passing the detection point at any instant, in absence of reaction, is known.

### *Gradient calibration procedure and equations*

Both titration procedures proposed are based on the concentration profile established in the FI manifold after the introduction of a discrete volume of sample (in SIT) or titrant (in TIT). Therefore, the first step necessary to both procedures is the calibration of this concentration gradient. This procedure is made, in the absence of any reaction, by using only one solution. Any solution containing a specimen capable of being sensed by the detector and producing a response linearly related with its concentration can be used at this stage. However, for simplicity, the titrant solution is usually employed. The left-hand side of figure 2 shows the signals obtained in the calibration process for the conductimetric detection. Initially, the titrant (NaOH in figure 2) is continuously pumped through its tubing until a steady-state conductimetric signal is obtained. At this point, all of the other tubes in the FI manifold contain water or conditioning

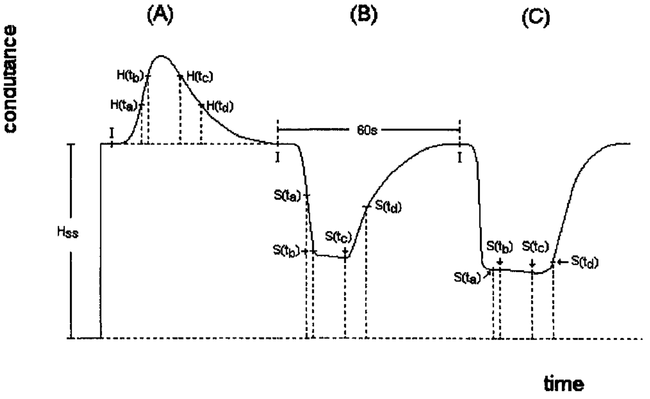

*Figure 2. (A) Gradient calibration signals; (B) and (C), signals for two consecutive (lower concentration on the left) conductimetric determinations of acetic acid in vinegar by the SIT. $H_{ss}$, detector signal obtained by pumping the titrant (NaOH) through its line until a steady-state is obtained; I, injection times; $H(t)$, height of the gradient calibration signal; $S(t)$ height of the titration signal Only four $H(t)$ and $S(t)$ points (from the 600–800 values actually obtained) are shown.*

(non-reactive) solutions. The height of this signal is denoted as $H_{ss}$ and it embodies the effect of the dilution suffered by the pumped solution due to confluence with other solutions found in its path to the detector.

The constant concentration (mol. $l^{-1}$) of the continuously pumped solution ($C$) passing the detection point, is given by:

$$C = \frac{q}{q_{total}} \cdot C^o \qquad (1)$$

where $C^o$ is the original solution concentration, $q$ is its pumping flow rate and $q_{total}$ is the total flow rate.

For a linear response the values of $q$ and $q_{total}$ are directly proportional to $H_{ss}$ and $H_{max}$, respectively. $H_{max}$ denotes the steady-state signal that would be obtained if the solution was pumped through all the tubing in the FI manifold. As will be shown below, the $H_{max}$ value does not need to be measured. Therefore, the value of $C$ can be also given by the expression:

$$C = \frac{H_{ss}}{H_{max}} \cdot C^o \qquad (2)$$

The same titrant solution is then injected in the manifold, carried by water, merged with the line containing the same solution and with the other pumping lines, passed through the mixing chamber, and the peak-like concentration profile, observed in figure 2a, is obtained over the steady-state signal ($H_{ss}$).

The data acquisition software is triggered when the solution is injected in the FI manifold and stores a number between 600 and 800 point $H(t)$ (a time dependent signal value, taken above the steady-state plateau to the peak profile signal). The $t$ index refers to the point collected and can be associated with the time elapsed after injection. Data collection starts at injection and is spaced for a fixed time interval of 110 ms. The height, $H_{ss}$, and the profile points, $H(t)$, are stored in order to be used in the construction of the titration curves. These data are also employed to find out the time dependent factors that convert the time elapsed from the instant of the injection of a solution in the manifold, to the instantaneous concentration of the specimens, present in that solution, at the detection point. For any solution injected in a given manifold, the instantaneous concentration (mol. $l^{-1}$) at the detector, $C(t)$, can be found by:

$$C(t) = g(t) \cdot C^o \quad (3)$$

where $C^o$ is the original concentration of the injected solution and $g(t)$ is the time dependent factor found by:

$$g(t) = \frac{H(t)}{H_{max}} \quad (4)$$

If necessary, a solution of titrant at lower concentration than the one injected during the determination, can be used to generate a lower $H_{ss}$ (steady-state signal) in order to keep the calibration signals in the linear range of the detector response.

Because the dispersion is caused by convection inside the mixing chamber, the $g(t)$ and $C(t)$ values are independent of the specimen being injected [8]. Therefore, the gradient calibration can be used by both the proposed methodologies as long as the titrant (in SIT) or sample (in TIT) is flowing through the same tube used to find the $H_{ss}$ value during the gradient calibration procedure.

*FI titration procedures*

The system is generally ready to perform a SIT just after the gradient calibration procedure is finished (which does not require more than 2 min), because the titrant is already being pumped through its tube at this stage. To perform a TIT procedure, the sample must replace the titrant or any solution in the tubing previously used in the gradient calibration and be pumped for a time interval long enough to wash out any solution previously present in that tube.

Samples or titrant solutions (depending on the titration procedure chosen) are injected under computer control and the resulting signals, now obtained in the presence of the reaction between the analyte and the titrant, are sampled in the same time intervals employed in the gradient calibration procedure. The right side of figure 2 (*b* and *c*) shows two typical SIT conductance ($S(t)$) × time curves obtained by injecting vinegar samples and reacting them with a continuously pumped sodium hydroxide solution.

*FI titration comportment*

Because it is possible to measure the analyte and titrant concentrations at the detection point on the SIT procedure then, in the absence of any reaction, the concentration × time curves in figure 3(*a*) should be obtained. The peak-like curve *S* is related to the analyte and curve *T* to the titrant. Four time intervals in the SIT procedure are important: the elapsed time when the concentration gradient of the analyte increases and there is an excess of the titrant ($t_1$ to $t_2$); when there is an excess of the

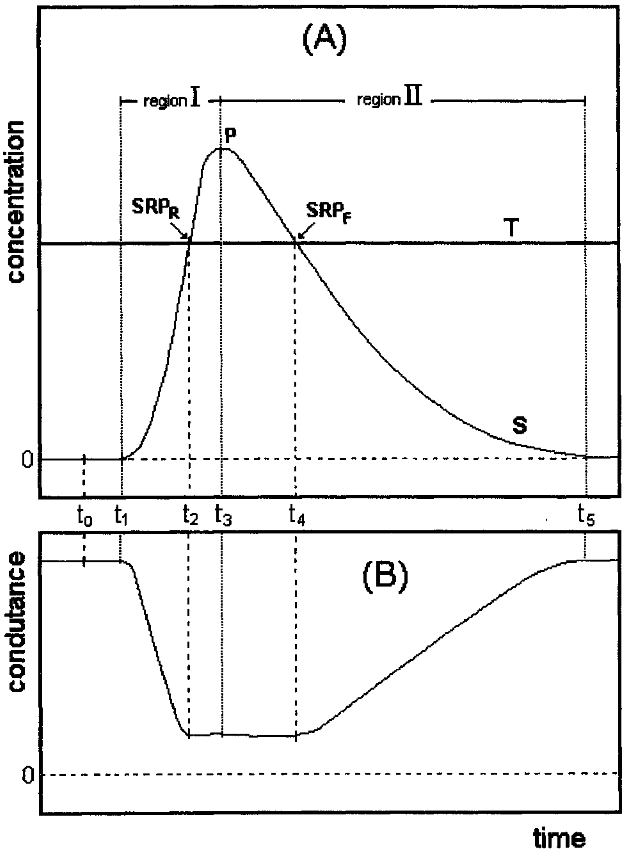

*Figure 3. (A) The hypothetical concentration × time for the SIT. (B) SIT curves for acetic acid determination. The peak-like curve S is related to the analyte and curve T to the titrant. The sample is injected at $t_o$, achieves the detection point at $t_l$, maximum concentration (point P) at $t_3$ and quits the detection point at $t_5$. The $SRP_R$ (at $t_2$) is the stoichiometric point related to rising region (region I) and $SRP_F$ (at $t_4$) related to the falling region (region II).*

analyte, the concentration gradient of the analyte increases and reaches the maximum ($t_2$ to $t_3$); when the analyte concentration gradient starts to decrease, but the concentration is still in excess ($t_3$ to $t_4$) and, finally, when the titrant returns to being in excess and the zone sample quits the detection point ($t_4$ to $t_5$).

Because the concentration of the injected solution first increases region *I* in (figure 3*a*), reaches a peak (*P* in figure 3*a* and decreases (region II in figure 3*a*), two SRP points are obtained: one related to the region which is rising, $SRP_R$, where the excess of the titrant is followed by an excess of analyte; and the other is related to the falling region, $SRP_F$, where excess of analyte is followed by an excess of titrant. The curve related to rising region shows an abrupt change in concentration × time profile and is not useful for the analytical procedure, because the $SRP_R$ cannot be located with reasonable precision. On the other hand, the curve related to the falling region clearly shows a smooth change in the concentration × time profile and is useful for the analytical procedure, because the $SRP_F$ can be located with good precision.

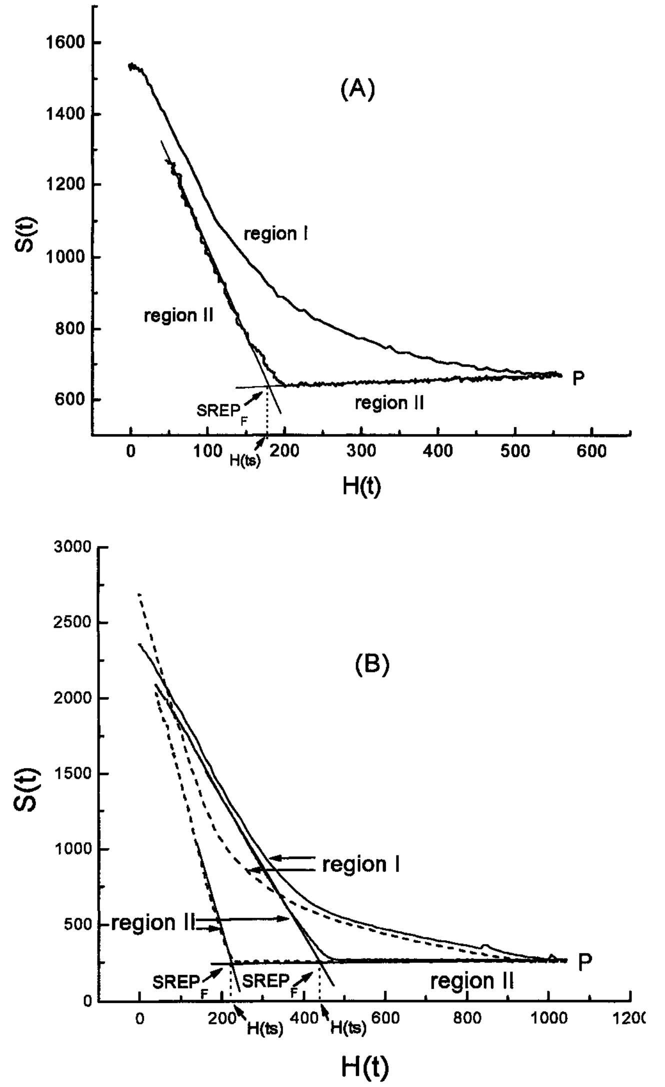

*Figure 4. (A) $S(t) \times H(t)$ (both in the arbitrary units of conductance) curve for conductimetric determination of acetic acid in vinegar (sample 1 in table 2) by SIT, showing the two straight lines used for $SREP_F$ and $H(ts)$ determination. (B) $S(t) \times H(t)$ (both in the arbitrary units of absorbance) curves for spectrophotometric determination of Fe(II) (samples: SS-4 (dashed curve) and SS-2 in Table 1) by the SIT. Regions I and II are the portions of the signal for the rising and falling concentration of the injected sample gradient; respectively. P is the signal related to the peak of the gradient calibration profile.*

In the TIT procedure a similar pattern is obtained, but the peak-like curve $S$ is related to the titrant and curve $T$ is related to the analyte.

In both the SIT or TIT procedures, the concentration of the injected solution, analyte (in SIT) or titrant (in TIT), at the interval time of the $t_2$ to $t_4$ (figure 3*a*), must be higher than the concentration of the solution continuously pumped, analyte (in SIT) or titrant (in TIT). If this condition is not fulfilled, the $SRP_R$ and $SRP_F$ will not be reached and the SIT or TIT will be incomplete.

In presence of a reaction, the curve reflects the change in the property being observed due the formation of the products. For example, the reaction between the acetic acid injected and NaOH continuously pumped causes the conductance of the resulting solution to decrease ($t_1$ to $t_2$ in figure 3*b*) as a consequence of the water formation, while the NaOH is in excess ($t_1$ to $t_2$ figure 3*a*). When the $SRP_R$ is achieved (at $t_2$), the conductance is at its minimum and subsequent excess of acetic acid ($t_2$ to $t_4$, figure 3*a*) causes a little change in the conductance ($t_2$ to $t_4$ in figure 3*b*). In the $SRP_F$ (at $t_4$), the conductance begins to increase ($t_4$ to $t_5$ in figure 3*b*) because the NaOH concentration is in excess in relation to the acetic acid concentration ($t_4$ to $t_5$ in figure 3*a*). The profiles shown in figures 2*b* and 2*c* are then obtained. Similar curves are obtained for the spectrophotometric determination of Fe(ii) by the SIT.

### *Conversion of the signal × time experimental curves to signal × H(t) curves*

The signal × concentration curves can be easily derived by replacing each point, obtained at any time interval after injection, for its equivalent $H(t)$ value obtained by the gradient calibration procedure. The $H(t)$ values are directly proportional to the concentration of the injected specimen. Figure 4 shows the effect of converting (in SIT) a $S(t) \times$ time to a $S(t) \times H(t)$ plot. The resulting plot (figure 4*a*) shows the behaviour of the signal as the analyte (acetic acid) concentration changes inside the mixing chamber feed with a constant sodium hydroxide concentration, as imposed by the flow system.

Figure 4*b* shows two $S(t) \times H(t)$ plots obtained for the spectrophotometric determinations of iron(II) by the SIT. The resulting plot reflects the change of instantaneous concentration of $KMnO_4$, since the spectrophotometer is monitoring the absorbance of the $KMnO_4$ at 525 nm.

### *Determination of stoichiometric ratio experimental points (SREPs)*

The SREPs employed to calculate the analyte concentration could, in principle, be located in the signal × time experimental curves obtained by any of the procedures (TIT or SIT) described above. However, these points can be located more precisely and the real behaviour of the analytical signal can be observed by using signal × concentration curves. It is not so easy to locate SREPs in the original signal × time curve as the nonlinear addition of the titrant or analyte with time, provided by the FI gradient, produces a very short linear portion after the SREP has been reached.

The SREP can be located in the $S(t) \times H(t)$ at the crossing point of the two straight lines observed after and before the abrupt change in the conductance, as shown in figure 4*a*, or in the absorbance as shown in figure 4*b*.

### *Analyte concentration determination*

After the SREPs have been found from the data analysis, the analyte concentration can be determined. The following reaction between the analyte ($A$) and the titrant ($T$) is assumed:

$$xA + yT \leftrightarrow A_xT_y$$

In the SREP, $t = ts$ and the $H(ts)$ associated with the SREP is found in the $S(t) \times H(t)$ curve and employed in the following calculations.

*Analyte concentration calculation for the SIT*

For a SIT, the instantaneous analyte concentration at the SRP ($C_A(ts)$) will be, in the absence of reaction, and using equations 3 and 4:

$$C_A(ts) = \frac{H(ts)}{H_{max}} \cdot C_A^o \quad (5)$$

where $C_A^o$ is the unknown analyte concentration in the sample solution.

The titrant concentration (in the absence of reaction) is constant during the titration and it is calculated as follows:

$$C_T = \frac{H_{ss}}{H_{max}} \cdot C_T^o \quad (6)$$

where $C_T^o$ is the original known concentration of the titrant present in its flask. At the SRP, the following relationship is valid:

$$y \cdot C_A(ts) = x \cdot C_T \quad (7)$$

Therefore, the original concentration of the analyte in the injected solution ($C_A^o$) can be obtained from equations 5, 6 and 7 and expressed by:

$$C_A^o = \frac{x \cdot H_{ss} \cdot C_T^o}{y \cdot H(ts)} \quad (8)$$

*Analyte concentration calculation for the TIT*

For the TIT procedure, the analyte concentration ($C_A$) is constant and is calculated as follows:

$$C_A = \frac{H_{ss}}{H_{max}} \cdot C_A^o \quad (9)$$

At the SRP, the following relationship is valid:

$$y \cdot C_A = x \cdot C_T(ts) \quad (10)$$

The instantaneous titrant concentration at the SRP is calculated by:

$$C_T(ts) = \frac{H(ts)}{H_{max}} \cdot C_A^o \quad (11)$$

Therefore the analyte concentration is:

$$C_A^o = \frac{x \cdot H(ts) \cdot C_T^o}{y \cdot H_{ss}} \quad (12)$$

Equations 8 and 12 show that the $H_{max}$ value does not need to be measured in order to establish the original concentration of the analyte ($C_A^o$).

To perform the calculations, the program searches for the $H(ts)$ values in the $S(t) \times H(t)$ curves. These values are always found at the crossing points of straight lines coming from two regions where the titrant and the analyte are in excess. The concentration of the titrant ($C_T^o$) needs to be known in order to find the analyte concentration. The titrant solutions ($KMnO_4$ or NaOH) were always standardized in this study.

*Table 1. Results, expressed in mmol. $dm^{-3}$, for the determination of Fe(II) in synthetic samples(SS), iron alloys(IA) and iron ores(IO) by the SIT procedure employing spectrophotometric detection. $KMnO_4$ concentration = 2·40 mmol. $dm^{-3}$.*

| *Sample* | *Classical titration* | *SIT** |
|---|---|---|
| SS-1 | 19·5 | 19·5 (±0·6%) |
| SS-2 | 35·5 | 36·0 (±1·2%) |
| SS-3 | 50·0 | 49·3 (±0·6%) |
| SS-4 | 64·9 | 65·5 (±1·1%) |
| SS-5 | 90·2 | 90·3 (±1·7%) |
| IA-IPT12A | 27·3 | 26·8 (±1·2%) |
| IA-IPT29 | 27·6 | 26·6 (±0·9%) |
| IA-IPT24 | 19·7 | 19·2 (±1·2%) |
| IO-IPT21 | 19·3 | 19·4 (±1·4%) |

* ±Relative standard deviation for 10 replicates.

### Results and discussion

*Results for the SIT*

The SIT results obtained for the concentration of synthetic samples of Fe(II) using $SREP_R$ were not good because the curve related to rising region of the peak (region *I* in figure 4b) did not give a clear $SREP_R$. This was because during the $t_1$ to $t_2$ stage (figure 3a), the Fe(II) concentration rose due to an excess of $KMnO_4$ titrant and species of Mn of intermediate oxidation state between Mn(VII) and Mn(II) were formed, changing the reaction stoichiometry. The falling region reflects the inverse and a clear $SREP_F$ was obtained (region *II* in the figure 4*b*). During the stage $t_3$ to $t_4$ (figure 3), there was an initial excess of iron(II) which decreased as it washed out from the mixing chamber until the concentration of the $KMnO_4$ was in excess. With an excess of Fe(II), Mn(VII) is directly converted to Mn(II) (colourless), causing the absorbance at 525 nm to decrease linearly and a stoichiometric reaction to be obtained.

Table 1 shows the results obtained for the determination Fe(II), by SIT, of five synthetic samples (SS1–SS5), three alloys (IA-IPT12A, IA-IPT29 and IA-IPT24) and one iron ore (IO-IPT21) using $SREP_F$. About 40 samples can be processed per hour by the spectrophotometric SIT procedure and the results agree with the classical titration.

The SIT procedure was also applied to conductimetric determination of acetic acid in vinegar. The results obtained for $SREP_R$ are not useful because no sharp transition in the conductance was obtained (see region I in figure 4a). However, a very good precision was obtained for the determination using $SREP_F$ (see table 2). This was the result of a well-defined transition in conductance. The system is capable of processing 60 samples per hour and the results also agree with those from classical titrations.

*Results for the TIT*

The TIT results obtained for the concentration of synthetic samples of Fe(II) using $SREP_F$ were also questionable. The curve $S(t) \times H(t)$ related to the falling region did not present a clear $SREP_F$. The same problem

*Table 2. Results, expressed in mmol. $dm^{-3}$, obtained for the determination of acetic acid in vinegar samples by the SIT procedure employing conductimetric detection. NaOH concentration = 0·100 mol. $dm^{-1}$.*

| Sample | Classical titration | SIT* |
|---|---|---|
| 1 | 804 | 796 (±0·7%) |
| 2 | 798 | 790 (±0·6%) |
| 3 | 828 | 831 (±0·4%) |
| 4 | 748 | 751 (±0·7%) |
| 5 | 866 | 874 (±0·8%) |

* ±Relative standard deviation for 10 replicates.

was obtained in the attempts to find the $SREP_R$ in the determination Fe(II) by the SIT. On the other hand, in the rising region of the gradient, the $SREP_R$ was achieved with a gradual addition of $KMnO_4$ over a fixed Fe(II) concentration of an Fe(II) solution which was continuously pumped. Therefore, at this stage, the Mn(VII) was directly converted to Mn(II) and a sharp $SREP_R$ was observed.

In the Fe(II) determination by the TIT using $SREP_R$, an average relative standard deviation and average relative error greater than 3% and 2%, respectively, were obtained. The precision of these results are not good due to a high fluctuation found in $H(ts)$ values. On the other hand, in the Fe(II) determination by the SIT, the average relative standard deviation (*c.* 1%) is lower than that for the TIT procedure, because the SREPs employed are found in the falling region of the concentration gradient profile, where the values for $H(ts)$ are less affected by small fluctuations in time because the concentration gradient is less abrupt.

### *Comparison between both procedures, analytical requirements and care*

Whether the SIT or TIT methodology should be used needs to be evaluated in terms of the specific application. If the quantity of sample is not a restrictive factor and if in-line monitoring is wanted, the TIT procedure is more economical as only small volumes of titrant are employed to perform each determination. On the other hand, the SIT procedure is more suitable when quantity is limited. In some cases, however, the procedure to be used may be dictated by the characteristics of chemical reaction involved in the titration. For example, the SIT procedure appears to be more suitable for the spectrophotometric determination of Fe(II), by using $KMnO_4$ solution as titrant, because a good precision is obtained for the SREP found at the falling region of the gradient, where the reaction is performed with slow decrease of the analyte concentration over that of the titrant solution. Side reactions are avoided by adding the Fe(II) solution to the $KMnO_4$ solution.

For both procedures, and when it is possible to choose, the SREP located at the falling region of the concentration profile must be employed for final calculations. However, because of the dynamic characteristic of the flow titration system, the SREP should not be located in the region of the falling gradient where very slow change in the concentration is observed, because the change in the signal may not be sharp enough to permit the precise localization of the SREP.

Routine determinations, made on samples whose concentration range is limited and previously known, could be further optimized by forcing SREPs to be located in the best region of the falling gradient. This region corresponds to the one where an almost linear change in concentration is observed and where standard deviations for the SREPs lower than 1% are easily achieved. The control over this parameter is made, in FI systems, by an appropriate selection of the titrant concentration and of the injected volume.

An overall comparison of the results obtained by the proposed flow technique and the classical titration reveals a relative accuracy of about 1%. The proposed procedures are reliable only if the concentration gradient profile, calibrated by the injection of the titrant, is equal to that produced by the sample (in SIT) or by the titrant (in TIT). The mixing chamber present in FI manifolds provides for this equality because the mass transport, that rules the dispersion, is by convection rather than by diffusion.

A directly proportional response of the detector in relation to the titrant concentration is required for the gradient calibration procedure, and a fast chemical reaction between the analyte and titrant must occur. Classical batch titration also benefits from the use of fast reactions, because the determination can be carried out quickly and accurately, although this is not essential because the batch procedure is not as time restrictive as the flow method.

### Conclusion

The possibility of using the concentration gradients, generated by FI systems, to perform analytical procedures similar to titrations and based on stoichiometric concentration ratios has been demonstrated. The instantaneous concentration of the analyte or titrant at the detection point was achieved employing the gradient calibration technique [14–16]. The SIT and TIT procedures proposed are, along with the binary searching method recently described [13], capable of employing stoichiometry to establish the analyte concentration in a flow system. Obviously, as in any automatic batch titration procedure made with the titrant being added at constant flow rate, the reaction between the analyte and titrant must be fast in order to be processed in the short time interval during which the specimens reside inside the mixing chamber.

The most attractive aspect of the proposed methodologies is the use of only one standard solution for calibration. The proposed methodologies are more suitable for continuous automatic monitoring than other flow procedures [3–7, 10–12], which require the use of various standard solutions for system calibration. Furthermore, the instantaneous concentration of titrant and analyte that defines the SRP is known. This is another characteristic that distinguishes the proposed flow methodology from others [3–7, 9–12] in which the concentration of titrant or analyte that defines the SRP was not known or employed.

The TIT works in a similar way to the flow rate gradient titration methodologies previously proposed [3–5]. However, the generation of the titrant gradient without changing the flow rate of the peristaltic pump is an advantage in terms of simplicity of the flow manifold, because it can be constructed with only one of these devices.

The methodologies proposed here require, as suggested by IUPAC definition of titration [21], the use of only one titrant whose concentration is well known and the stoichiometric ratio experimental points are located between regions where excess of titrant or analyte occurs. The advantages of classical titration procedures are retained by the suggested procedures. The detector needs to be stable only during the gradient calibration procedure while the relationship between time and concentration is being established. After this procedure has been completed, small fluctuations will not significantly affect the location of SREPs.

### References

1. Ziegel, H., *Fresenius Z. Anal. Chem.*, **20** (1914), 285.
2. Cunha, I. B. S. and Pasquini, C., *Analyst*, **117** (1992), 905.
3. Blaedel, W. J. and Laessig, R. H., *Analytical Chemistry*, **36** (1964), 1617.
4. Ashworth, M. R. F., Welish, W., Becker, W. and Stutz, F., *Fresenius Z. Anal. Chem.*, **273** (1975), 275.
5. Abitch, S. M., *Analytica Chimica Acta*, **114** (1980), 247.
6. Fleet, B. and Ho, A. Y. W., *Analytical Chemistry*, **46** (1974), 9.
7. Nagy, G., Fehér, Z., Tóth, K. and Pungor, E., *Analytica Chimica Acta*, **91** (1966), 87.
8. Ruzicka, J. and Hansen, E. H., *Flow Injection Analysis* (Wiley, New York, 1988).
9. Ruzicka, J., Hansen, E. H. and Mosbaek, H., *Analytica Chimica Acta*, **92** (1977), 235.
10. Clark, G. D., Zable, J., Ruzicka, J. and Christian, G. D., *Talanta*, **38** (1991), 119.
11. Astrom, O., *Analytica Chimica Acta*, **105** (1979), 67.
12. Ishibashi, N. and Imato, T., *Fresenius Z. Anal. Chem.*, **323** (1986), 244.
13. Korn, M., Gouveia, L. F., de Oliveira, E. and Reis, B. F., *Analytica Chimica Acta*, **313** (1995), 177.
14. Araújo, M. C. U., Pasquini, C., Bruns, R. E. and Zagatto, E. A. G., *Analytica Chimica Acta*, **171** (1985), 337.
15. Pasquini, C., Araújo, M. C. U. and Bruns, R. E., *Laboratory Microcomputer*, **9** (1990), 44.
16. Silva, E. C., Araújo, M. C. U., Honorato, R. S., Costa Lima, J. L. F., Zagatto, E. A. G. and Brienza, S. M. B., *Analytica Chimica Acta*, **319** (1996), 153.
17. Basset, J., Denney, R. C., Jeffrery, G. H. and Mendham, J., *Vogel's Textbook of Quantitative Inorganic Analysis* (Longman, London, 1978).
18. Araújo, M. C. U., Honorato, R. S., Santos, A. V. and Silva, E. C., *Química Nova*, **19**, (1996), 86.
19. Pasquini, C. and de Faria, L. C., *Journal of Automatic Chemistry*, **4** (1991), 143.
20. Malcome-Lawes, D. J., Pasquini, C. and Wong, K. H., *Laboratory Microcomputer*, **8**, (1989), 44.
21. Fraiser, H. and Nancollas, G. H., *Compendium on Analytical Nomenclature, Definitive Rules 1987* (Blackwell, Oxford, 1987).

